# Iron Deposition Is Positively Related to Cognitive Impairment in Patients with Chronic Mild Traumatic Brain Injury: Assessment with Susceptibility Weighted Imaging

**DOI:** 10.1155/2015/470676

**Published:** 2015-12-20

**Authors:** Liyan Lu, Heli Cao, Xiaoer Wei, Yuehua Li, Wenbin Li

**Affiliations:** ^1^Department of Radiology, Shanghai Jiao Tong University Affiliated Sixth People's Hospital, No. 600 Yi Shan Road, Shanghai 200233, China; ^2^Department of Neurosurgery, Shanghai Jiao Tong University Affiliated Sixth People's Hospital, No. 600 Yi Shan Road, Shanghai 200233, China

## Abstract

*Background*. This study aimed to evaluate the usability of SWI in assessment of brain iron to detect cognitive dysfunction in patients with chronic mild traumatic brain injury (mTBI).* Methods*. 39 patients with mTBI and 37 normal controls were given the Mini-Mental State Examination (MMSE) and underwent SWI scanning at least 6 months after injury. Angle radian values were calculated with phase images. The angle radian values were compared between groups using analysis of covariance, and their association with MMSE scores was analyzed using Spearman correlations.* Results*. Significantly higher angle radian values (*p* < 0.05) were found in the head of the caudate nucleus, the lenticular nucleus, the hippocampus, the thalamus, the right substantia nigra, the red nucleus, and the splenium of the corpus callosum (SCC) in the mTBI group, compared to the control group. MMSE scores were negatively correlated with angle radian values in the right substantia nigra (*r* = −0.685, *p* < 0.001).* Conclusions*. Patients with chronic mTBI might have abnormally high accumulations of iron, and their MMSE scores are negatively associated with angle radian values in the right substantia nigra, suggesting a role of SWI in the assessment of cognitive impairments of these patients.

## 1. Introduction

Traumatic brain injury (TBI) is a major and serious public-health concern throughout the world [1]. The majority of TBIs are mild TBI (mTBI), which is referred to as concussion, according to the Committee of the Head Injury Interdisciplinary Special Interest Group of the American Congress of Rehabilitation [2]. Approximately 15% to 30% of patients complain of an array of cognitive symptoms following mTBI [3, 4]. If the cognitive symptoms do not end within 3 months, they may persist throughout life.

In most mTBI cases, cognitive impairment is nonspecific and CT or MRI shows normal structure; therefore, patients might be underestimated, leading to long-term disabilities in their work and social interactions. Therefore, potential noninvasive, advanced MRI methods that can contribute to the prediction of cognitive dysfunction in patients with mTBI are demanded.

Susceptibility weighted imaging (SWI) which has the potential to provide the increased sensitivity needed to detect and characterize lesions is a high-resolution structural MRI technique. It uses a sequence that is sensitive to the presence of iron and blood products and functional blood oxygenation changes [5–12] in the brain to reveal the magnetic susceptibility changes between tissues [6, 13]. Studies to date have demonstrated detecting the brain iron with the help of SWI can be useful for detecting Alzheimer's disease (AD) and amnestic mild cognitive impairment (aMCI) [14, 15]. Meanwhile, there is evidence that iron becomes a source of pathology after mTBI throughout a number of metabolic mechanisms, including the generation of reactive oxygen species (ROS), the exacerbation of oxidative stress from other sources, and the formation of neurofibrillary tangles [16–18]. Thus, SWI might be useful for detecting TBI-related accumulation of iron and estimating the degree of cognitive impairment in mTBI.

As the relationship between brain iron and cognitive impairment in patients in the chronic stage of mTBI remains poorly understood, this study was to examine whether the usability of SWI in assessment of brain iron could detect cognitive dysfunction in patients with chronic mild traumatic brain injury. We hypothesized that angle radian values can be associated with cognitive impairment in order to establish the clinical utility of SWI as a prognostic biomarker of patients suffering cognitive dysfunction after mTBI.

## 2. Methods

### 2.1. Ethics Statements

The study was approved by the Institutional Review Board of Shanghai Jiao Tong University Affiliated Sixth People's Hospital and it was performed in accordance with the Declaration of Helsinki. All patients gave written informed consent.

### 2.2. Participants

#### 2.2.1. TBI Participants

Patients with mTBI (*N* = 46) were enrolled in a prospective cohort. Participants were recruited in the emergency room (ER) immediately after injury between 2011 and 2014. Inclusion criteria were (i) age 18 to 67 years; (ii) the presence of a closed head injury; (iii) mTBI evaluated initially at an emergency room (Glasgow Coma Score [GCS] of 13–15), loss of consciousness <20 min, posttraumatic amnesia <24 h, and a negative clinical MRI scan (without SWI) and no neurologic deficits; and (iv) persistent cognitive deficits after mTBI diagnosed by a trained neuropsychologist during the clinical evaluation of the patient's symptoms. Exclusion criteria were (i) hospitalization after the head injury; (ii) brain abnormalities on MRI scan; (iii) history of preexisting neurological or psychiatric disease; (iv) history of illicit drug use or substance abuse; and (v) previous head injury or cognitive impairment. However, the data from seven participants are not collected successfully. Thus, the data from 39 participants are reported.

#### 2.2.2. Control Participants

The control participants (*N* = 37) were recruited through advertisements. All of the control subjects underwent the same neuroimaging protocol as the patients did. The control participants met the same exclusion criteria applied to the patient group.

Patients and healthy control participants who were left-handed were excluded.

### 2.3. Cognitive Function Examination

Cognitive function was determined by Chinese language version of the Mini-Mental State Examination (MMSE) [19, 20], which included 30 items. The maximum score of MMSE is 30, with higher scores indicating better cognitive function. The widely accepted cut-off score of cognitive impairment in China (Chinese cut-off of MMSE, CCM) is education-specific: 17/18 for illiteracy, 20/21 for people with 0–6 years of education, and 24/25 for people with more than 6 years of education [19, 20]. Commonly used MMSE cut-off worldwide was 23/24 [19, 20]. In this study, we analyzed the data by applying both standards.

All of the participants underwent a research MRI scan at least 6 months after injury (19.37 ± 7.88 months). Participants completed neurologic and cognitive tests (the MMSE) at that time. Demographic characteristics and outcome measurement of mTBI and healthy control groups are presented in Table 1.

### 2.4. Imaging Protocol

The images were acquired on a 3T MR Scanner (MAGENTOM, Verio, Siemens Healthcare, Erlangen, Germany) using a 32-channel head coil. The imaging sequences consisted of T1W, T2W, diffusion weighted imaging (DWI), and fluid-attenuated inversion recovery (FLAIR) and SWI. The scanning parameters were as follows: T2W: axial scanning TR/TE, 6000 ms/95 ms; flip angle, 150°; an image matrix, 384 × 384; the slice thickness, 6 mm; distance factor, 30%; average, 1; and field of view (FOV), 250 mm; T1W fluid-attenuated inversion recovery: axial and sagittal scanning TR/TE, 2000 ms/9 ms; flip angle, 150°; matrix, 320 × 320; slice thickness, 6 mm; distance factor, 30%; average, 1; and FOV, 250 mm; and SWI: axial scanning TR/TE, 28 ms/20 ms; flip angle, 15°; matrix, 320 × 320; slice thickness, 1.2 mm; average, 1; and FOV, 230 mm. T2W used Turbo Spin Echo (TSE) sequence and SWI used 3D gradient echo (GRE) sequence. We employed the high-pass filter to remove the low-spatial frequency components of the background field. The “corrected” phase image is used to create a “phase” mask that is used to multiply the original magnitude image to create novel contrasts in the magnitude image [21]. Thus, a group of magnitude, phase, maximum intensity projection (MIP), and SWI images were automatically reconstructed [22, 23].

### 2.5. Image Analysis

**Figure 1:**
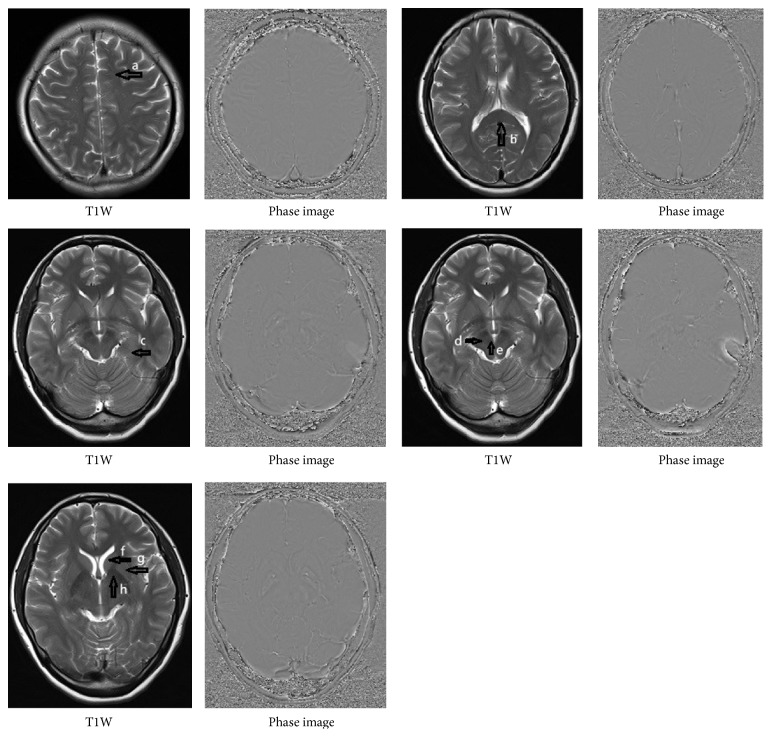
Representative locations of the ROIs (arrows) on axial T1W images and corresponding phase images: (a) lobe of white matter; (b) splenium of the corpus callosum (SCC); (c) hippocampus; (d) substantia nigra; (e) red nucleus; (f) head of caudate nucleus; (g) lenticular nucleus; (h) thalamus.

Angle radian values were measured with the phase image. Two neuroradiologists with more than 5 years of experience were blind to patients' clinical details that manually outlined the head of the caudate nucleus, the lenticular nucleus, hippocampus, thalamus, substantia nigra, red nucleus, the genu of corpus callosum (GCC), splenium of the corpus callosum (SCC), white matter of the frontal lobe, and the cerebellum as the regions of interest (ROIs) based on T1W and magnitude images. The ROIs were drawn in a single slice where they were best seen. Their size was adapted to the size of the structure, but the borders of the structures were excluded to avoid partial volume effects [24]. ROIs selected were copied to SWI and phase images by using the “copy boundary” function with SPIN (signal processing in nuclear magnetic resonance) software. All images were analyzed within 3 weeks by the same person to ensure there were similar ROI selections through all patients [24]. Representative locations of the ROIs on T1W images and corresponding phase images were shown in Figure 1. We obtained the mean radian angle values for these ROIs. The mean values were then calculated for statistical analysis. As the measured value (*X*) had a range of (−4096 to 4095), which mapped to the phase value *Y* (Pi to −Pi), the formula used for conversion was as follows: *Y* = (−*X* × *π*)/4096 [23], where *X* is the direct measured value on the phase image and *Y* is the corresponding angle radian value (phase value of the radius). The detailed processing steps have been described previously by Wang et al. [22, 23].

### 2.6. Data Analysis

Analysis of covariance and correction for multiple comparisons by using Levene's Test for Equality of Variances were performed in the study to compare differences between patients with mTBI and the controls in terms of regional angle radian values, adjusted for age. The relationship of the angle radian values with the MMSE scores was evaluated using Spearman correlations. A *p* value less than 0.05 was used as the criterion of statistical significance for all of the analyses.

## 3. Results

### 3.1. Angle Radian Values between mTBI and Control Groups

The means and SDs of the angle radian values in patient and control groups are summarized in Table 2. Compared with control group, significantly higher angle radian values in the head of the caudate nucleus (left: *p* < 0.001; right: *p* < 0.001), the lenticular nucleus (left: *p* < 0.001; right: *p* < 0.001), the hippocampus (left: *p* < 0.05; right: *p* < 0.001), the red nucleus (left: *p* < 0.05; right: *p* < 0.001), the right substantia nigra (*p* < 0.001), and the SCC (*p* < 0.005) were obtained in patient group. Representative phase images of SWI from eight brain sections of one patient with mTBI are shown in Figure 1.

### 3.2. Correlation between Angle Radian Values and MMSE Scores

The cognitive scores of patients group are reported in Table 1. The mean ± SD MMSE score in the patients was 25.21 ± 1.76. MMSE scores were negatively correlated with angle radian values in the right substantia nigra (*r* = −0.685, *p* < 0.001) in the mTBI group.

## 4. Discussion

Between 10% and 20% of individuals experience persistent cognitive symptoms in the chronic stage of mTBI [3]. In mTBI, identified morphologic changes are barely detectable by conventional neuroimaging techniques (computed tomography (CT) and magnetic resonance imaging (MRI)), probably due to the subtle extent and nature of mTBI lesions; obvious disruption of structure does not necessarily occur [25]. Moreover, mTBI lesions evolve over time due to a metabolic cascade of events. Therefore, their persistent symptoms are difficult to treat based on conventional clinical imaging.

Diffusion tensor imaging (DTI) has been considered as a biomarker for mTBI, as white matter (WM) microstructural alteration can be visualized. Two reports [4, 26] described DTI in TBI patients with cognitive impairment. A decrease in fractional anisotropy (FA) and an increase in mean diffusivity (MD) in TBI patients may correlate with axonal degradation [25, 27]. However, these and most studies of DTI have examined moderate to severe TBI patients close to the time of injury. In a recent study of “mTBI,” the reported brain hemorrhages of the participants suggest that more severe injuries may have occurred [2]. An earlier report [28] on mTBI included a subgroup with older brain injuries, but cognitive dysfunction was not involved. Although a number of DTI researches have investigated brain abnormalities, they yield inconsistent findings [2, 4, 25–28].

Patients with mTBI who have cognitive impairment usually show decreased spontaneous brain activity on a functional MRI (fMRI) [29–31]. fMRI is a considerably valuable tool in investigating and identifying the neuroanatomical substrates of cognitive disorders and monitoring their treatment [30], but fMRI is a complicated method.

Recent animal and human studies have implicated abnormal iron in the pathogenesis of the neurodegenerative disorders [17, 18, 32–34]. Iron plays a critical role in the cognitive impairment of the human brain. Whether the cognitive symptoms associated with the chronic stage of mTBI are attributable to elevated iron deposition in patients remains unknown.

A signal strength method was employed to detect brain iron, using T2W^*∗*^ low signal intensity image classification in past studies [35, 36]. In contrast, SWI sequencing has a higher resolution and a higher signal to noise ratio (SNR) and requires less time and produces more accurate measurements [37]. SWI could show magnetic susceptibility differences between ferrous and nonferrous tissues [15] which could enhance our ability to detect, evaluate, and monitor iron overconcentration diseases [13, 15].

In the present study, increased angle radian values were observed, using SWI, in a number of regions, including the head of the caudate nucleus, the lenticular nucleus, the hippocampus, the red nucleus, the substantia nigra, and the SCC, which suggests that iron might be a source of pathology in mTBI in line with a small body of studies [32–34].

We found that angle radian values changes were most predominantly in gray matter (GM), a result that is consistent with previous reports [16, 17], suggesting not only varied patterns of WM and the gray-white matter junction abnormalities in the mTBI patients, but also subcortical GM abnormalities, due to its central location in the brain.

Recent DTI studies of animals and humans have demonstrated that increased FA in GM is linked to prolonged symptoms [4]. GM regions are sensitive to traumatic damage because of the long fibers that originate or pass through them [3, 17, 38, 39] and because they participate in communication among sensory, motor, and associative areas [17, 40]. Thus, damage to GM structures can cause widespread cognitive impairments [41].

Increased angle radian values in mTBI could indicate excessive iron deposition to some extent. In vivo iron plays an essential role in the metabolic processes as a cofactor for numerous proteins. Brain iron abnormalities belong to two categories of physiologic iron: nonheme iron and heme iron [16]. Nonheme iron is associated with abnormal or dysfunctional iron transport pathways. The authors have postulated that the degeneration of neurons may result in the release of free iron [33]. It also has been shown that trauma-induced increased blood-brain barrier permeability [17, 42] and the phagocytosis of red blood cells can lead to the focal deposition of the heme iron. However, animal studies have shown that mTBI causes subtle axonal damage and oxidative stress injuries that release free radicals [43, 44]. When free radicals accumulate and the pH is lowered, the conditions are favorable for the deposition of iron [17, 45], which suggests another mechanism for excessive iron deposition in mTBI.

Abnormal iron deposition can be injurious to the brain and brain systems, as iron is a transitional metal and participates in redox reactions to form reactive oxygen species (ROS) that, in turn, can cause oxidative stress [16]. In addition, a pathologic case study [46] of a patient with mTBI who died unexpectedly reported a considerable amount of hemosiderin-laden macrophages in the frontal lobe, though both the CT results and the gross macroscopic postmortem evaluation were negative [17]. This is in line with findings in experimental models of brain trauma in which diffuse brain hemosiderin deposits were present even when there was no gross detection of hemorrhage [17, 47] and with the demonstrated improvement in spatial memory performance in animal models of trauma after treatment with deferoxamine, an iron scavenger [17, 48].

MMSE scores in the present study were negatively correlated with angle radian values in the right substantia nigra in the mTBI group, suggesting that cognitive impairment might be related to abnormal accumulation of iron. The substantia nigra is involved in reward and addiction. But now, there has been evidence of iron storage in the substantia nigra which is involved in cognitive impairment as well such as spatial memory performance. Meanwhile, the brain iron content is increased in the substantia nigra pars compacta (SNc) of PD patients; further, this area is known to have abnormal brain iron level in a host of pathological conditions, such as AD [24, 49].

We did not find significant increases in angle values in the thalamus, the white matter of frontal lobe, and the left substantia nigra. This can be partly examined by the fact that lower accumulation of iron was revealed in the examined areas [17]. The sensitivity of SWI to detect brain iron would decrease if the iron content is too low. In addition, the white matter injury is more dependent on the site of head injury, which varied in our patients [17]. Meanwhile, if the brain iron is too high, the brain iron may be underestimated by using the SWI sequence and analytic software [35] (Haacke et al., 2010). The main limitation of this study is the small number of participants, which makes it difficult to draw conclusions about the relationship between iron deposition and cognitive functioning. Although SWI is not a new way to measure susceptibility, SWI has exquisite capability to highlight anatomical structures which contain iron in comparison with other techniques.

## 5. Conclusions

This is the first study to investigate the correlation between brain iron and cognitive impairment among patients in the chronic stage of mTBI using SWI. The study found that patients in the chronic stage of mTBI have multiple regions of increased angle radian values, including the head of the caudate nucleus, the lenticular nucleus, the hippocampus, the red nucleus, the substantia nigra, and the SCC. The increased angle radian values in the right substantia nigra are strongly implicated in being related to persistent cognitive impairments in patients with chronic mTBI. In conclusion, this study suggested a role of SWI for the estimation of cognitive impairment of mTBI patients in the chronic stage.

## Figures and Tables

**Table 1:** Comparison of the patient cohort with study population.

	Patients (*n* = 39)	Healthy controls (*n* = 37)
Age (yr), mean (SD)	38.54 ± 13.15	38.51 ± 13.21
Male : female	22 : 17	19 : 18
Initial GCS (range)	14.08 ± 0.84 (13–15)	
Time after injury (mo)	19.37 ± 7.88	
MMSE	25.21 ± 1.77	

**Table 2:** Comparison of angle radian values in 15 brain regions between mTBI group and control group.

	mTBI		Controls		*F* value	*p* value
	Mean	SD	Mean	SD		
Brain region						
L, head of caudate nucleus	−3.4848	0.8224	−2.406	0.8223	480.453	<0.001^*∗*^
R, head of caudate nucleus	−2.513	0.8223	−1.93	0.4298	111.287	<0.001^*∗*^
L, lenticular nucleus	−2.7601	1.0171	−1.93	0.4298	124.104	<0.001^*∗*^
R, lenticular nucleus	−2.513	0.8223	−1.401	0.624	1255.25	<0.001^*∗*^
L, happocampus	−0.9892	2.2719	−0.704	1.721	4.397	0.039^*∗*^
R, happocampus	−1.1788	1.0905	−0.601	0.632	25.886	<0.001^*∗*^
L, thalamus	−0.515	1.4037	−0.45	1.4112	0.096	0.745
R, thalamus	−0.3258	1.8679	−0.227	1.9001	0.098	0.755
L, substantia nigra	−0.5931	1.6514	−0.488	1.5949	1.033	0.313
R, substantia nigra	−0.9918	2.2027	−0.366	1.6056	12.351	<0.001^*∗*^
L, red nucleus	−2.3264	2.2036	−1.954	1.5068	5.014	0.028^*∗*^
R, red nucleus	−2.6523	1.5869	−1.806	1.065	79.547	<0.001^*∗*^
Splenium of the corpus callosum (SCC)	0.9557	1.9476	0.915	1.4037	8.458	0.005^*∗*^
L, white matter of frontal lobe	−0.2259	1.9091	−0.326	1.8679	0.1	0.753
R, white matter of frontal lobe	−0.1476	1.895	−0.275	1.8745	0.095	0.759

Note: asterisk indicates statistically significant comparisons.
